# Stereotactically Guided Microsurgical Approach for Deep-Seated Eloquently Located Lesions

**DOI:** 10.3390/jcm14124175

**Published:** 2025-06-12

**Authors:** Jun Thorsteinsdottir, Sebastian Siller, Biyan Nathanael Harapan, Robert Forbrig, Jörg-Christian Tonn, Tobias Greve, Stefanie Quach, Christian Schichor

**Affiliations:** 1 Department of Neurosurgery, LMU University Hospital, LMU Munich, 81377 Munich, Germany; 2 Department of Neurosurgery, University Hospital Regensburg, 93053 Regensburg, Germany; 3 Institute of Neuroradiology, LMU University Hospital, LMU Munich, 81377 Munich, Germany; 4 Department of Neurosurgery, Evangelisches Klinikum Bethel, Bielefeld University, 33617 Bielefeld, Germany

**Keywords:** cavernoma, stereotactic neurosurgery, deep-seated, eloquent, intraoperative computed tomography, iCT, brain shift, neuronavigation, stereotaxy

## Abstract

**Background/Objectives**: Advancements in neuronavigation and intraoperative imaging have made gross-total resection of deep-seated lesions more feasible. However, in eloquently located regions, brain shift can lead to unintentional damage of functionally critical tissue during the approach. This study analyzes the feasibility and outcomes of a stereotactically guided microsurgical approach supported by intraoperative CT (iCT) for such lesions. **Methods**: Patients with deep-seated, eloquently located lesions treated between 03/2017 and 04/2023 at the Department of Neurosurgery, Ludwig-Maximilians-University (LMU) Munich, Germany, were included. Frame-based, image-guided stereotaxy was used for trajectory planning and catheter placement, verified by iCT. Microsurgical resection was conducted along the catheter trajectory using 2 mm conical blade retractors and continuous neurophysiological monitoring. Postoperative MRI assessed the extent of resection. Neurological outcomes were evaluated postoperatively, at 6 weeks, and at long-term follow-up in 12/2023. **Results**: A total of 12 patients were treated using the stereotactically guided microsurgical approach described in this study. In all cases, the implanted catheter precisely matched the preoperative trajectory, as confirmed by fused iCT data. Median durations were 23 min for stereotaxy and 3 h 7 min for microsurgery. Complete resection was achieved in all cases. One patient experienced transient hemiparesis and aphasia, both of which were fully resolved. All other patients showed neurological improvement or remained seizure-free at long-term follow-up. **Conclusions**: In selected cases, a stereotactically guided microsurgical approach with iCT enabled intraoperative localization of the target with high spatial accuracy and without immediate procedure-related complications in this limited cohort. Our findings support the feasibility of the technique; however, conclusions regarding clinical efficacy or broader applicability are limited by the small sample size and non-comparative study design.

## 1. Introduction

Modern neurosurgical technical armamentarium, including neuronavigation, ultrasound and intraoperative MRI or CT, allows a safe resection of deep-seated lesions in the majority of cases. However, in selected cases, especially small lesions located in the basal ganglia, thalamus or mesencephalon, microsurgical approaches can be challenging. The pitfalls are (1) parenchymal damage caused by localized pressure of retractor systems, (2) white matter tract dissection in functionally relevant locations and (3) aberrancies of the trajectory in the depth [1,2,3,4]. Localized pressure of retractor systems could lead to perioperative morbidity, including venous infarction and cerebral edema, resulting in focal neurological deficits or seizures [5,6]. Additionally, in cases of small lesions, spatial disorientation—particularly in depth—can increase the risk of inadvertently affecting functionally important brain areas during surgery. Therefore, technical advances in minimally invasive approaches, including tubular brain retraction and endoscopy-assisted surgeries, have been introduced to create a safer and smaller trajectory [7,8,9,10,11]. Advanced multimodal neuroimaging has been established to be incorporated into neuronavigation for minimizing trajectory aberrancies in depth [12,13,14]. Diffusion-tensor-imaging (DTI) fibertracking has been proven to be useful for planning the surgical approach for deep-seated lesions adjacent to the corticospinal tract [12,13]. Furthermore, intraoperative image guidance by intraoperative ultrasound navigation has improved depth orientation, enhancing surgical precision [15]. However, these technical features are not available in every neurosurgical center. A stereotactic approach offers a precise alternative for targeting deep-seated eloquent lesions while minimizing functional risk. In our center, we have developed significant expertise in stereotactic procedures, characterized by a very low rate of perioperative morbidity and reduced surgical time [16]. Stereotaxy is of advantage compared to neuronavigation in providing a trajectory with very high accuracy, avoiding brain shift caused by craniotomy and subsequent loss of cerebrospinal fluid (CSF). Especially in deep-seated eloquent regions, minor errors in the angulation of the approach could induce severe neurological deficits [17]. While stereotactic procedures are commonly used to obtain small tissue samples, they typically do not facilitate the resection of lesions.

In consideration of the benefits and limitations of the mentioned techniques, we sought to optimize the surgical strategy for resection of deeply-seated eloquently located lesions by combining frame-based stereotaxy—to define the approach by positioning a stereotactically placed catheter with its tip pointing at the lesion—and frameless microsurgery for removal of the lesion. This study aims to investigate the surgical and neurological outcome in a series of selected patients harboring deep-seated lesions located in functionally relevant areas using this combined stereotactically catheter-guided microsurgical approach.

## 2. Materials and Methods

### 2.1. Patient Selection and Study Design

All procedures involving human participants adhered to the ethical standards set by the institutional and/or national research ethics committees, as well as the 1964 Declaration of Helsinki and its subsequent revisions or equivalent ethical guidelines. Informed consent was obtained from all participants and/or their legal representatives. The study received approval from the Institutional Review Board of Ludwig-Maximilians-University Munich (Project #18-098) and complied fully with its regulations concerning informed consent.

We conducted a single-center retrospective study to investigate the clinical and surgical outcomes in all consecutive cases operated upon with this technique between March 2017 and April 2023 (Table 1).

Demographic information, case records, radiological images and operative outcomes were analyzed. All patients received pre-operative MRI for stereotaxy and neuronavigation (GE signa HDx 3T scanner), including T1, T2, T2 *, FIESTA images (1 mm slice thickness), contrast-enhanced T1, MR angiography and diffusion-tensor-imaging. Postoperative CT scan was performed on day 1 after surgery. Extent of resection was assessed by MRI during in-hospital stay (except for one patient who suffered from a pulmonary embolism and received a CT). Deviation of intraoperative catheter position was matched with postoperative MRI using neuronavigation, and deviation was calculated in 3 points along the catheter. Craniotomy size, trajectory length and trajectory diameter were measured using iPlan 3.0 Cranial (Brainlab, Munich, Bavaria, Germany). Craniotomy size was assessed on postoperative CT, trajectory length was determined based on the implanted catheter and trajectory diameter was measured on postoperative MRI in axial, coronal and sagittal slices. Postoperative clinical outcome was assessed daily during hospitalization and after six weeks. Clinical outcome was assigned by modified Rankin score (mRS) and Glasgow Outcome Scale (GOS). Long-term follow-up with a particular focus on seizure outcomes was performed in 12/2023.

### 2.2. Surgical Technique

**Figure 1:**
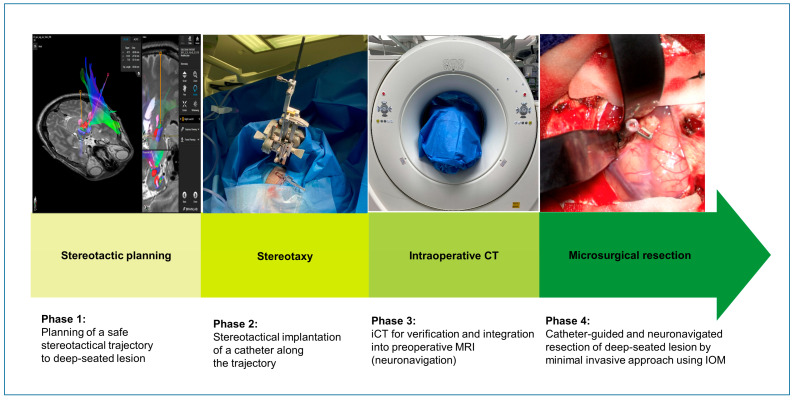
Workflow of the stereotactic catheter-guided neuronavigated approach. Phase 1 shows the stereotactical planning of a safe trajectory to the deep-seated, eloquently located lesion. Phase 2 demonstrates the stereotactical implantation of the catheter targeting the lesion. Phase 3 illustrates intraoperative CT for verification of the correct catheter location. Phase 4 shows the stereotactic guided approach along the implanted catheter to the lesion.

The protocol for surgical technique is illustrated in Figure 1. Frame-based multimodal image-guided stereotactic technique was used for trajectory planning connecting the cortical entry point and the surface of the lesion. For each patient, an optimal trajectory to the lesion was generated, avoiding contact with arterial or venous vessels and maintaining a minimal distance of 2 mm between the trajectory and any vessel. Subsequently, a catheter targeting the lesion was stereotactically implanted using this trajectory. Stereotactically localized CT angiography (0.6 mm slice-spacing, 1.46 mSv) was fused with preoperative MRA, T1- and T2-weighted MRI data. Trajectories were planned using Target@1.19 software (Brainlab, Munich, Bavaria, Germany). The catheter (1.3 mm ventricular catheter, Medtronic, Minneapolis, MN, USA) was implanted through a 5 mm skin incision, a 3 mm diameter burr hole, and secured with a microclip at dura level, and skin was closed using 4.0 prolene suture. After removal of the stereotactic frame, the patient was positioned on a carbon-made radiolucent surgical table (TruSystem 7500, Trumpf Medical, Saalfeld, Thüringen, Germany). The patient’s head was fixed in radiolucent head clamp (Mayfield, A-2002, Integra, Princeton, NJ, USA) [18], and a high-resolution unenhanced cranial CT (0.6 mm slice spacing, 1.05 mSv) was acquired using iCT to confirm the correct catheter position. ICT (Siemens SOMATOM Definition AS+, Siemens Healthineers, Munich, Bavaria, Germany) was obtained by a ”double room CT scanner“, as described earlier [19,20,21]. ICT data set was fused with preoperative multimodal imaging data, including diffusion-tensor-imaging using iPlan 3.0 Cranial (Brainlab, Munich, Bavaria, Germany). After an approximately 3 × 3 cm craniotomy, a 1.5 × 1.5 cm durotomy around the catheter and corticotomy of 5 mm, the minimally invasive approach was continued microscopically along the surface of the catheter. Retractors with conical blades with a 2 mm diameter (DORO^®^, Black Forest Medical group, Freiburg, Baden-Württemberg, Germany) were used for preparation. The trajectory was followed by standard navigation system during the approach to visualize potential brain shift. During resection, continuous intraoperative monitoring (IOM), including somatosensory (SEP) and motor evoked potentials (MEPs), was recorded in all cases as described previously [22]. Once the lesion was located, the stereotactically implanted catheter was removed and the lesion was resected. In case of cavernous malformations, we first bluntly entered the lesion with small, fine-tip bipolar forceps to evacuate blood and decompress the lesion. The cavernoma was then carefully separated from the surrounding tissue using suction and microdissectors, and in most cases, it was removed in a piecemeal manner. Hemostasis was performed with a long bayonet-style fine bipolar (SilverGlide Keyhole Maximum Access, Stryker, Fremont, CA, USA) and oxidized regenerated cellulose (Tabotamp, Ethicon Co. for Johnson & Johnson Medical, Piscataway, NJ, USA). Dural and craniotomy closure were performed as usual.

## 3. Results

### 3.1. Patient Characteristics

Among 1927 patients harboring supratentorial tumors and 93 patients harboring supratentorial cavernomas, a total of 0.6% of patients (2 male, 10 female) with deep-seated and/or highly eloquent located cavernous malformations were operated using this stereotactically guided microsurgical approach. Characteristics of patients and lesions are given in Table 2. Median age was 36.5 years (range: 17.5–66.5 years). Lesions were located on the right/left hemisphere in 7/5 patients, and in the basal ganglia in three patients (25%), cella media in two patients (16.7%), thalamus in one patient (8.3%), insula in one patient (8.3%), superior temporal gyrus in two patients (16.7%), cerebral peduncle in one patient (8.3%), trigonum in one patient (8.3%) and splenium in one patient (8.3%). Initial symptoms were focal seizures in six patients (50.0%), hypesthesia in five patients (41.6%), aphasia in three patients (25%) and hemiparesis in two patients (16.7%). The median size of the lesion was 20 mm (range: 11–31 mm), and the median depth of the lesion from the cortical surface was 42 mm (range: 10–62 mm). Histology revealed cavernous malformation in ten patients (83.3%), pilocytic astrocytoma (8.3%) in one patient and meningioma in one patient (8.3%).

### 3.2. Surgical Results Using the Stereotactically Guided Neuronavigated Microsurgical Approach

Lesion- and surgery-associated parameters are presented in Table 3. Median craniotomy size was 35 mm (range: 27–45 mm), median trajectory length was 62.7 mm (range: 43.2–72.1 mm) and mean trajectory diameter was 6.0 mm ± 1.2 mm (range: 4.2–9.4 mm) in postoperative MRI. The median duration of stereotaxy was 23 min, and the mean duration of microsurgery was 3 h 7 min. Median blood loss was 100 mL (range: 30–300 mL), and median hospital stay was 7 days (range: 6–15 days). After stereotactic target achievement by catheter implantation, the skin was closed, and iCT confirmed the correct placement of the catheter targeting the lesion. A standard microsurgical approach was then initiated using intraoperative neuronavigation with ultrasound as a backup system to confirm the expected brain shift. However, the implanted catheter pointed with its tip at the lesion in all cases and was not affected by brain shift. Complete removal of the lesion was achieved in all patients verified by postoperative MRI. In one case, a patient with a cavernoma located in the splenium was first operated by a standard microsurgical approach. As the lesion was not entirely removed in the postoperative MRI, the patient was successfully operated on using the stereotactic approach (Supplementary Figure S1). Postoperatively, no cases of epidural, subdural or intraparenchymal bleeding were observed. There were no cases in which this method was unexpectedly deemed unsuitable during the operation. Moreover, there were no instances where conversion to a standard microsurgical approach involving a larger craniotomy was necessary. One adverse event occurred in one patient suffering from pulmonary embolism on the first postoperative day, which was treated with anticoagulation.

### 3.3. Neurological Outcome

Neurological outcome in our patient cohort is shown in Table 1. Immediately after surgery, eight symptomatic patients (mRS score = 1) presented without any neurological deficit (mRS score = 0). One asymptomatic patient with a cavernoma in the basal ganglia and one asymptomatic patient with a meningioma in the trigonum remained without any neurological deficit. One patient with a cavernous malformation in the left thalamus suffered from transient right hemiparesis (2/5) and hypesthesia in the right arm (mRS score = 3). Postoperative CT did not reveal any haemorrhage or infarction. The symptoms were attributed to perioperative manipulation in close vicinity to the corticospinal tract. Until discharge, symptoms resolved completely and were not detectable in neurological examination at the last FU (mRS score = 0). One patient with a partially cystic pilocytic astrocytoma located in the thalamus, cerebral crus and internal capsule, who was pretreated with interstitial brachytherapy, suffered preoperatively from left hemiparesis (mRS score = 3). Immediately after surgery and until the last FU, hemiparesis improved from initially 3/5 proximal and 1/5 distal to proximal 5/5 and distal 3/5 (mRS score = 2).

In total, after a median follow-up time of 34.5 months (range: 4.5–52.0 months), 11 patients did not show any neurological deficit and one patient with preoperative hemiparesis improved neurologically. One patient had one focal seizure after cessation of anticonvulsive therapy but remained asymptomatic until the last FU. None of the patients showed any new deficit, and all patients were seizure-free at long-term follow-up.

### 3.4. Case Report

**Figure 2:**
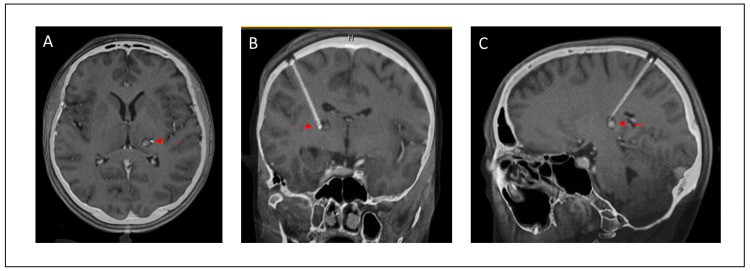
Fusion of the preoperative T1-weighted MRI and intraoperative CT. The preoperative contrast-enhanced T1-weighted MRI sequence in axial (**A**), coronal (**B**), and sagittal (**C**) reconstruction was fused with intraoperative CT showing the catheter which targets the lesion. Red arrows point to the tip of the stereotactically implanted catheter targeting the lesion.

**Figure 3:**
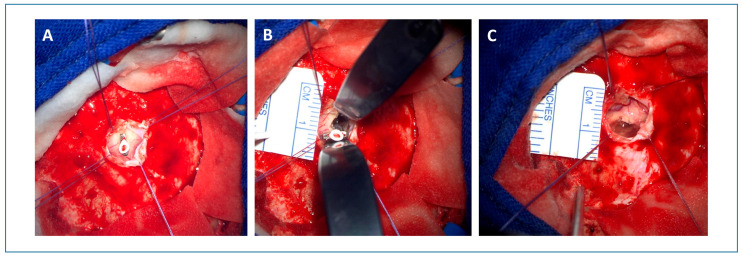
Microsurgical approach. A small craniotomy was performed, and the dura was opened around the stereotactically implanted catheter (**A**). Using a microscope, preparation with brain stem spatulas was performed along the stereotactically implanted catheter, and the trajectory was confirmed by neuronavigation (**B**). As soon as the lesion was found and confirmed by neuronavigation, the implanted catheter was removed and the lesion was thoroughly resected. The picture illustrates the corticotomy after removal of the lesion (**C**).

**Figure 4:**
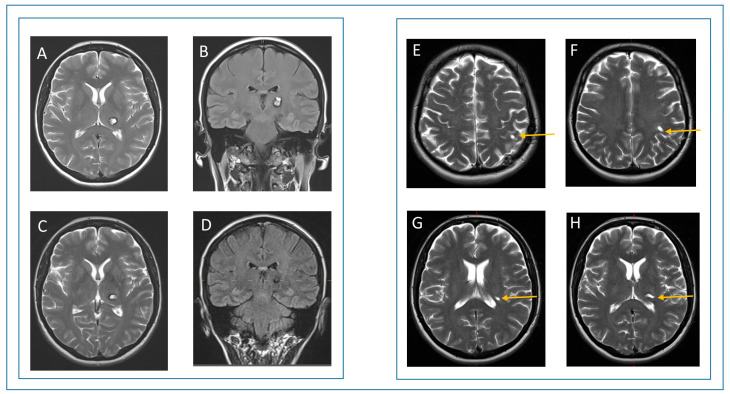
Pre- and postoperative MRI. On the left, the first row shows a cystic lesion in the left thalamus in T2 weighted sequence (**A**) and T2-FLAIR sequence (**B**). The second row shows a complete removal of the lesion. The hemosiderin ring is still slightly visible in T2-weighted sequence (**C**) and T2-FLAIR sequence (**D**). On the right, the postoperative course and size of the trajectory (yellow arrows) are shown in axial plane (**E**–**H**).

A 52-year-old female presented with acute hemihypesthesia in the right face and arm accompanied by a motor deficit of the right leg and a decrease in her fine motor skills in the right hand (patient #11, see Table 1 and Table 2). The symptoms resolved partially within a month. The first MRI revealed a partially hemorrhagic lesion suspicious of cavernous malformation in the left thalamus (Supplementary Figure S2). The patient was referred to our department for a second opinion after six months. Based on the evidence of space-occupying hemorrhage and the symptoms, the indication for surgery was set. Preoperative MRI showed a cystic, slightly contrast-enhancing lesion with previous hemorrhages in the left thalamus (Figure 2). A catheter targeting the lesion was stereotactically implanted. Post-implantation intraoperative CT confirmed a correct catheter position. Thereafter, a microsurgical approach was performed along the surface of the catheter (Figure 3). Intraoperative monitoring, including MEP and SEP, showed no abnormalities. Postoperatively, the patient presented with a transient hemiparesis on the right, which resolved completely after 7 days, including the pre-operative deficit. Postoperative CT showed neither new nor remaining hemorrhage. Control MRI after three months showed a complete removal of the cavernous malformation (Figure 4). At the last follow-up after 2 years, the patient did not show any neurological deficit.

## 4. Discussion

Recent technical innovations in microneurosurgery have significantly improved access to deep-seated cerebral lesions by enabling more effective and less invasive surgical corridors. Techniques such as transcortical approaches, cortical/subcortical electrical stimulation and awake surgeries have enhanced surgical safety and efficacy by tailoring the surgical corridor to the functional topography [23,24]. However, the approach to deep-seated, eloquently located lesions remains a considerable challenge.

Intraoperative brain deformations during surgery can lead to severe neurological deficits, especially in eloquent regions, where deviations of just a few millimeters can profoundly affect the patient’s neurological outcome. This precision is particularly crucial in procedures such as deep brain stimulation for Parkinson’s disease or stereoelectroencephalography for identifying epileptogenic foci, where therapeutic success depends on the accurate placement of electrodes in the correct anatomical targets [25,26]. It has been demonstrated that opening the dura mater can cause displacement of neural structure especially due to the loss of the physiological negative intracranial pressure, air inflow, and loss of CSF [25]. Reports show substantial variability in deformation errors ranging from 15 to 24 mm for cortical displacements [27,28] and 8 to 31 mm for tumor regions using neuronavigation-assisted microsurgery [29].

To address these challenges, we employed a stereotactically guided microsurgical approach that combines precise lesion localization within eloquent areas and allows bimanual resection of the lesion under intraoperative monitoring to preserve functional outcomes.

As early as the 1990s, Hassenbusch et al. used stereotactic markers to visualize the margins of brain tumors, primarily to delineate tumor borders in an era where intraoperative imaging was not available [30]. In the past decade, intraoperative imaging systems have received significant attention. In particular, intraoperative ultrasound (iUS) integrated with neuronavigation data has proven useful for real-time intraoperative representation of large, eloquently located tumors, assessing the extent of resection [31,32,33]. However, iUS is highly operator-dependent, challenging to standardize and may require repeated examinations to detect small deep-seated lesions [31,32,33]. Alternatively, intraoperative MRI (iMRI) provides valuable information on the extent of tumor resection, which is crucial for postoperative outcomes [27,34]. However, iMRI is time-consuming and not available in every neurosurgical center [27,34]. iUS, iMRI, and fluorophores such as 5-ALA or Indocyanine green (ICG) are among the most advanced tools for real-time intraoperative visualization. While these technologies are crucial for large, infiltrating lesions to assess the extent of resection, our stereotactic technique may be advantageous for maintaining the correct trajectory during dissection to small, well-circumscribed lesions without significant edema.

In our study, iCT was used for stereotactic catheter implantation and verification of the correct catheter position. To minimize radiation exposure, our neuroradiologists developed a new iCT protocol using an automated tube current modulation and voltage selection, reducing the radiation dose from 2.97 mSv to 1.46 mSv [35]. In the future, MRI-based stereotaxy may be a viable alternative to avoid radiation exposure.

We intentionally separated the stereotactic catheter implantation from microsurgical resection in order to minimize the risk of catheter dislocation during patient repositioning or craniotomy by employing the following individual measures: (1) stereotactic technique to maximize accuracy in targeting the lesion; (2) verification of correct catheter placement using intraoperative CT after removal of the stereotactic frame; (3) facilitating bimanual manipulation for lesion resection under intraoperative stimulation and monitoring and (4) adaptation of the aperture diameter/location in depth during resection. Importantly, we did not observe any catheter dislocation, as it was sufficiently secured.

In minimally invasive neurosurgery, several techniques have been developed to enhance surgical precision while reducing patient morbidity. Each approach offers distinct advantages and presents specific challenges. Awake surgery approaches allow for continuous monitoring of neurological functions during surgery with immediate adjustments based on patients’ responses, which can enhance neurological outcomes. Although awake surgeries do not demand specialized equipment, the necessity for a highly skilled multidisciplinary team can negate potential cost savings. Endoscopic or tube systems, combined with conventional microscopy, enable minimally invasive approaches to deep-seated lesions with improved illumination and magnification [7,8,9,11,36,37,38]. On the downside, many endoscopic systems may restrict instrument maneuverability, potentially complicating surgical procedures [7]. Therefore, surgeons may require specialized training to effectively use endoscopic equipment. Endoscopic systems and specialized instruments represent a financial investment, though they may be more cost-effective compared to the acquisition and maintenance of neuronavigation systems and intraoperative CT. Tubular retractor systems provide increased visualization of deep intra-axial sites, with minimal disruption to the functional tissue compared to traditional plate retraction systems [11,36,37]. Also, self-retaining retractors create a stable operative field, potentially reducing operative time. Depending solely on microscopic visualization through the tube may not provide comprehensive views of the surgical field. Also, similarly to endoscopic systems, the confined space within the tube can limit instrument movement and complicate lesion resection [9,11]. Due to low costs and simple technology, tubular retractor systems may be more accessible in various surgical settings.

In our study, we used a retractor system with conical blades (2 mm diameter), which simultaneously minimized the risk of iatrogenic tissue and vascular damage from brain retraction while allowing bimanual manipulation and adjustment of the aperture diameter in depth. With a mean size of 6.0 ± 1.2 mm, the trajectory diameter in postoperative MRI was smaller than endoscopic or tubular approaches with a mean cannulation diameter of 9 mm [7,36,37]. Although our procedure, including stereotaxy planning, intraoperative CT and microsurgical resection, required slightly more surgery time than former studies, no deviations from the planned trajectory or uncertainties of the intraoperative approach were encountered. A recent study involving five patients demonstrated the feasibility of resecting deep-seated arteriovenous malformations (AVM) using stereotactically guided tubular retractor systems [39]. However, the mean maximum AVM nidal diameter was 8.2 mm, limiting the procedures to smaller AVMs but with favorable neurological and surgical outcomes [39]. A comparison with other surgical techniques is illustrated in Table 4. In recent studies, augmented reality (AR) was able to improve surgical planning, refine neuronavigation and reduce operation time by overlaying computer-generated images onto the user’s real-world visual field. Especially with regard to the extent of resection, AR offers a better identification of tumor boarders. Also, AR enhances surgical planning for skull-base lesions by intraoperative navigation-integrated projections onto the surface of the skull [40]. However, studies for stereotactic procedures showed that AR-guided or ARN-assisted operations may be feasible for large targets as the accuracy error is still 1–3 mm, and most AR systems do not prevent accuracy loss due to brain shift [41].

Limitations of the study are the retrospective design and the small sample size as this was a practicality and feasibility study. Our study included 10 patients with cavernomas, 1 patient with pilocytic astrocytoma and 1 patient with a meningioma. Resection of the cavernomas and meningioma was more straightforward due to clear visualization and tactile differentiation from normal brain tissue compared to the pilocytic astrocytoma. Nonetheless, we acknowledge that lesion consistency, vascularity and border definition—which vary significantly between, for example, cavernomas and gliomas—could affect technical reproducibility and feasibility. The haptic differences among these lesions emphasize that while the technical access (trajectory planning, stereotactic guidance and microsurgical technique) remains constant, intraoperative adaptability based on tactile feedback is essential. We now note that reproducibility of this technique is likely highest in well-circumscribed lesions such as cavernomas or deep-seated, encapsulated tumors and may require further refinement or adaptation in other histologies.

Evidently, this technique necessitates specialized neurosurgical equipment for the operative workflow, including stereotaxy, intraoperative CT and microsurgery. Nevertheless, in experienced centers, this approach may be reasonable in selected cases. Given the potential for severe neurological deficits resulting from millimeter-scale deviation caused by brain shift, we believe that this approach combining frame-based stereotaxy and frameless catheter-guided microsurgery is justified. Importantly, this study should be understood as a feasibility study. The selected cohort represents a highly specific and narrowly defined subset of patients for whom the stereotactically guided microsurgical technique was judged to be particularly beneficial. We acknowledge the limitations that arise from this selective approach, including its impact on the generalizability of our findings. It is also important to clarify that this feasibility study does not demonstrate the superiority of the technique over other approaches, and no such claims should be inferred from the results. We believe that demonstrating safe and effective outcomes in this highly selected cohort provides valuable insights for other centers managing similarly challenging cases. These findings may also serve as a foundation for future prospective or comparative studies aimed at further evaluating the utility and generalizability of this approach. Further research with larger, more diverse cohorts is necessary to validate the efficacy and safety of this approach in a multicenter prospective study.

## 5. Conclusions

In summary, we implemented a frame-based multimodal imaging-guided stereotactic technique for trajectory planning and catheter implantation targeting the lesion. Simultaneously, intraoperative monitoring, including motor- and sensory-evoked potentials, enabled real-time assessment of neurological function during microsurgical resection of the lesion. In this small cohort, the technique enabled accurate trajectory planning with no intraoperative deviations documented and parenchymal disruption appeared limited based on postoperative imaging and clinical course. Neurological outcomes were favorable, and seizure status remained stable or improved in those with a history of epilepsy. This study demonstrates the feasibility of this stereotactically guided microsurgical approach in a highly selected patient cohort. While initial outcomes appear encouraging, conclusions regarding clinical efficacy are limited by the small, heterogeneous cohort and the non-comparative study design.

## Figures and Tables

**Table 1:** Inclusion and exclusion criteria for lesion selection.

Inclusion Criteria	Exclusion Criteria
- single lesion	- multiple lesions
- intraparenchymatous/intraventricular lesion	- no anatomical association to nerval structures
- well circumscribed in T1-weighted imaging with contrast enhancement	- infiltrative growth
- without significant perilesional edema (on T2-weighted imaging)	- perilesional edema (on T2-weighted imaging)
- 10–30 mm in diameter	- >30 mm in diameter
- ≥4 cm from the cortical surface AND/OR location in eloquent regions, e.g., basal ganglia, thalamus, mesencephalon or language-associated cortical areas	
- suspicion of a benign lesion	- suspicion of a highly aggressive tumor

**Table 2:** Patient characteristics and neurological outcomes.

Patient No.	Age	Gender	Side	Location	Preoperative Neurological Deficit	Preoperative mRS	Preoperative GOS	Postoperative Neurological Deficit	Postoperative mRS	Postoperative GOS	Neurological Deficit at Last FU	mRS, Last FU	GOS Last FU
1	43	F	L	white matter of the central region	focal seizures (facial numbness and spasms)	1	4	none	0	5	none	0	5
2	18	F	L	dorsal capsula interna, lentiform nucleus	focal seizures, ataxia, spasticity	1	4	none	0	5	none	0	5
3	16	F	R	thalamus, cerebral crus, dorsal internal capsule	left hemiparesis (proximal 3/5, distal 1/5), hemihypesthesia	3	3	left hemiparesis (proximal 4/5, distal 2/5), hemihypesthesia improved	2	4	left hemiparesis (distal 3/5), hemihypesthesia improved	2	4
4	66	M	R	frontal operculum	hemihypesthesia	1	4	none	0	5	none	0	5
5	59	F	R	basal ganglia	no deficit (incidental finding)	0	5	none	0	5	none	0	5
6	28	F	R	insula, external capsule, putamen	hemihypesthesia	1	4	none	0	5	one seizure after cessation of anticonvulsants	1	5
7	37	F	L	eloquent superior temporal gyrus	focal seizures (expressive aphasia)	1	4	none	0	5	none	0	5
8	27	F	L	eloquent superior temporal gyrus	focal seizures (expressive aphasia)	1	4	none	0	5	none	0	5
9	36	F	R	anterior part of the insula	focal seizures (hypesthesia right arm, dysarthria)	1	4	none	0	5	none	0	5
10	19	M	R	trigonum	no deficit (incidental finding)	0	5	none	0	5	none	0	5
11	52	F	L	thalamus	hypesthesia right arm	1	4	transient right hemiparesis (2/5), hypesthesia right arm	3	3	none	0	5
12	46	F	R	splenium	focal seizures	1	5	none	0	0	none	0	5

F, female; FU, follow up; L, left; M, male; R, right.

**Table 3:** Lesion- and surgery-associated parameters.

Patient No.	Lesion Size [mm]	Lesion Depth [mm]	Histology	Craniotomy Size [mm]	Trajectory Length [mm]	Mean Trajectory Diameter [mm]	Std Mean Trajectory Diameter [mm]	Duration of Planning and Stereotaxy [min]	Duration of Surgery [min]	Extent of Resection (in MRI)
1	18	43	Cavernoma	43	61.4	5.9	1.6	00:23	03:43	GTR
2	22	46	Cavernoma	45	62.7	4.9	1.0	00:34	04:00	GTR
3	31	62	pilocytic astrocytoma	34	64.5	6.8	2.5	00:23	05:42	GTR
4	19	41	Cavernoma	36	48.6	6.5	1.3	00:25	02:44	GTR
5	27	52	Cavernoma	33	66.6	4.7	1.5	00:15	03:14	GTR
6	23	36	Cavernoma	27	43.2	5.5	0.4	00:22	03:48	GTR
7	11	13	Cavernoma	32	45.7	7.0	0.6	00:23	02:16	GTR
8	22	14	Cavernoma	31	44.0	9.4	3.1	00:22	03:00	GTR
9	20	24	Cavernoma	36	68.8	4.2	0.6	00:19	02:53	GTR
10	18	36	Meningioma	30	66.8	6.1	0.4	00:46	03:25	GTR
11	10	46	Cavernoma	41	72.1	5.2	0.8	00:25	02:55	GTR
12	19	57	Cavernoma	19	63	5.1	0.3	00:32	01:57	GTR (after second surgery)

GTR, gross total resection; MRI, magnet resonance imaging; std, standard deviation.

**Table 4:** Comparison of our approach with recent neurosurgical techniques for deep-seated lesions.

Study (Year)	Approach	No. of Patients	Diameter [mm]	Median Size of Lesion [mm]	Median Craniotomy Size [mm]	Operative Time	Median Blood Loss [mL]	Complication Rate	Neurological Outcome	Extent of Resection
Liu et al. [38] (2022)	Neuronavigation-Guided Transcortical-Transventricular Endoport-Assisted Endoscopic Resection for Thalamic Lesions: Preliminary Experience	8	NA	31	NA	NA	NA	n = 2 (electrolyte disturbance) n = 1 (subdural hematoma) n = 1 (severe pneumonia) n = 1 (DVT)	Improved (n = 3) Stable (n = 3) Worsened (n = 2)	GTR (n = 4) NTR (n = 3) STR (n = 1)
Achey et al. [39] (2023)	Surgical Resection of Deep-Seated Arteriovenous Malformations Through Stereotactically Guided Tubular Retractor Systems: A Case Series.	5	NA	8.2	NA	NA	NA	NA	NA	GTR (n = 5)
Takeuchi et al. [37] (2022)	Efficacy and safety of the endoscopic “wet-field” technique for removal of supratentorial cavernous malformations	13	6 (n = 8) 10 (n = 4) 17 (n = 1)	22.9	55 (range: 40–70)	NA	NA	NA	Improved (n = 12) Stable (n = 1)	GTR (n = 12) STR (n = 1)
Our study	Stereotactically guided microsurgical resection of deep-seated or eloquently located lesions using intraoperative computed tomography	12	6 ± 1.2	19.5	35 (range: 27–45)	23 min/3 h 7 min	100 (range: 30–300)	NA	Improved (n = 1) No deficit (n = 11)	GTR (n = 12)

## Data Availability

Authors can confirm that all relevant data are included in the article. Dataset(s) derived from public resources and made available with the article (references).

## Supplementary Materials

The following supporting information can be downloaded at: https://www.mdpi.com/article/10.3390/jcm14124175/s1, Figure S1: Preoperative MRI, MRI after first surgery and MRI after second surgery (stereotactically guided approach); Figure S2: Initial MRI at diagnosis (case report).

## Abbreviations

The following abbreviations are used in this manuscript:

iCT	Intraoperative computer tomography
DTI	Diffusion-tensor-imaging
mRS	modified Rankin score
GOS	Glasgow Outcome Scale
CSF	Cerebrospinal fluid
iUS	Intraoperative ultrasound
iMRI	Intraoperative magnetic resonance imaging
GTR	gross total resection
STR	subtotal resection
NTR	near total resection
DVT	deep vein thrombosis
