# Supplementary material for: Stereotactically Guided Microsurgical Approach for Deep-Seated Eloquently Located Lesions
Source: J Clin Med. 2025 Jun 12;14(12):4175. doi: 10.3390/jcm14124175 (PMC12194318; doi:10.3390/jcm14124175)
Supplement: Supplementary file 1 [file jcm-14-04175-s001.zip › Suppl. Figure S1.pptx]

## Slide 1
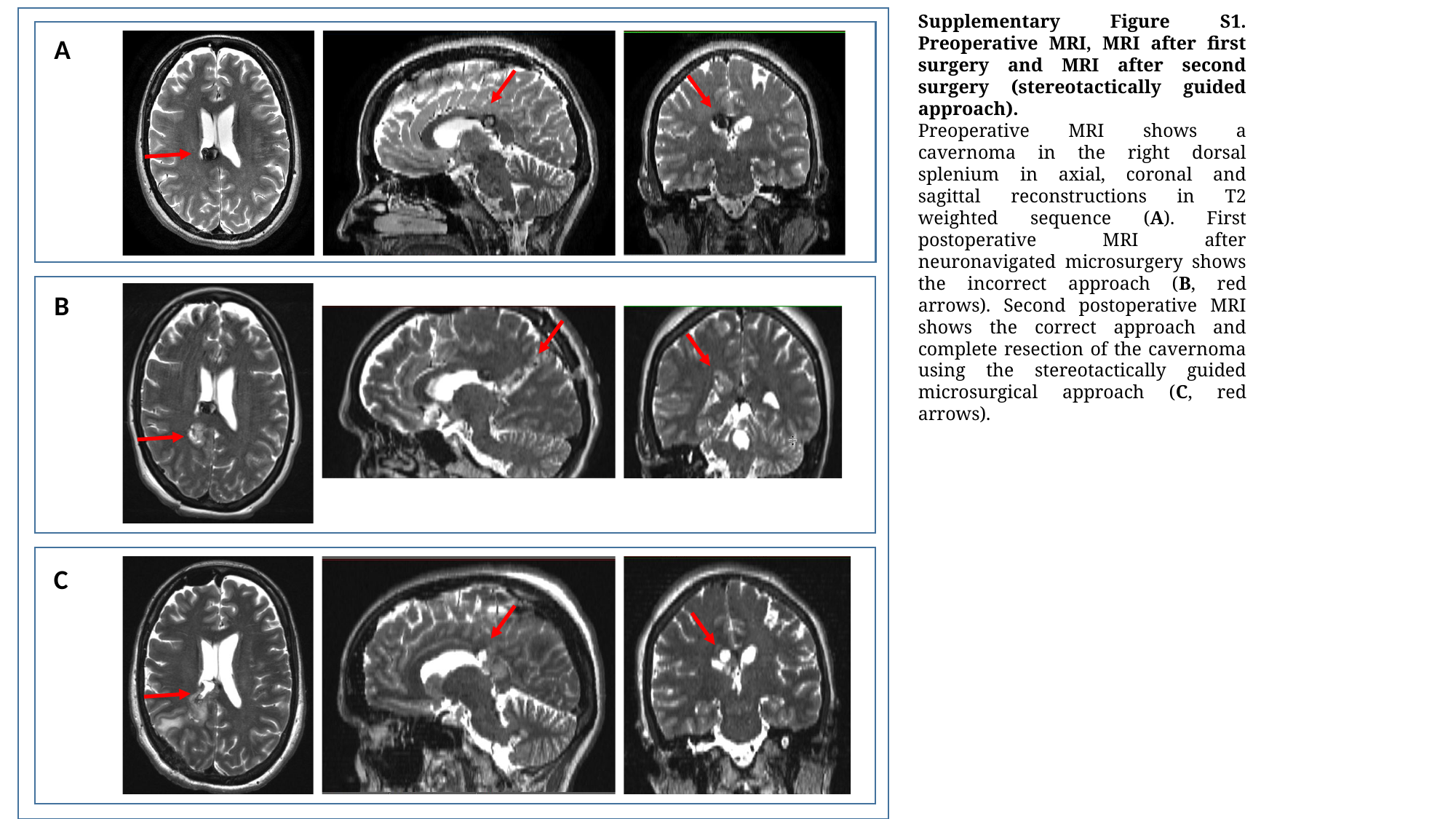

Supplementary Figure S1. Preoperative MRI, MRI after first surgery and MRI after second surgery (stereotactically guided approach).
Preoperative MRI shows a cavernoma in the right dorsal splenium in axial, coronal and sagittal reconstructions in T2 weighted sequence (A). First postoperative MRI after neuronavigated microsurgery shows the incorrect approach (B, red arrows). Second postoperative MRI shows the correct approach and complete resection of the cavernoma using the stereotactically guided microsurgical approach (C, red arrows).
A
B
C
