# Supplementary material for: Stereotactically Guided Microsurgical Approach for Deep-Seated Eloquently Located Lesions
Source: J Clin Med. 2025 Jun 12;14(12):4175. doi: 10.3390/jcm14124175 (PMC12194318; doi:10.3390/jcm14124175)
Supplement: Supplementary file 1 [file jcm-14-04175-s001.zip › Suppl. Figure S2.pptx]

## Slide 1
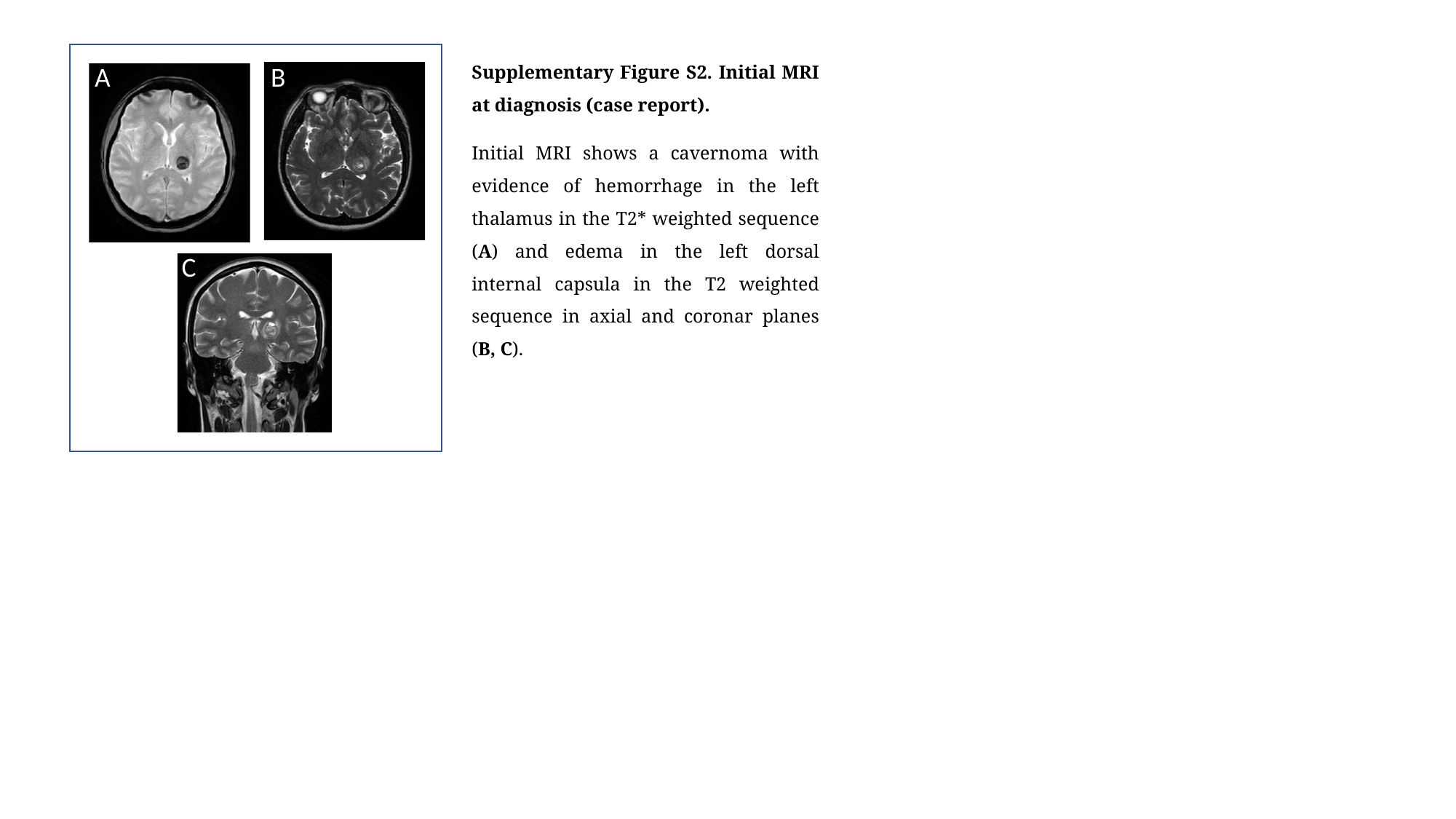

Supplementary Figure S2. Initial MRI at diagnosis (case report).
Initial MRI shows a cavernoma with evidence of hemorrhage in the left thalamus in the T2* weighted sequence (A) and edema in the left dorsal internal capsula in the T2 weighted sequence in axial and coronar planes (B, C).
A
B
C
